# Numerical Analysis of the Mitigation Performance of a Buried PT-WIB on Environmental Vibration

**DOI:** 10.3390/s23187666

**Published:** 2023-09-05

**Authors:** Lei Gao, Chenzhi Cai, Chao Li, Cheuk Ming Mak

**Affiliations:** 1 School of Civil Engineering, Central South University, Changsha 410000, China; l.gao@connect.polyu.hk (L.G.); chenzhi.cai@csu.edu.cn (C.C.); 2 Department of Building Environment and Energy Engineering, The Hong Kong Polytechnic University, Hong Kong

**Keywords:** environmental vibration, attenuation zone, periodic structure, wave impedance block, finite element

## Abstract

Environmental vibration pollution has serious negative impacts on human health. Among the various contributors to environmental vibration pollution in urban areas, rail transit vibration stands out as a significant source. Consequently, addressing this issue and finding effective measures to attenuate rail transit vibration has become a significant area of concern. An infilled trench can be arranged periodically along the propagation paths of the waves in the soil to attenuate vibration waves in a specific frequency range. However, the periodic infilled trench seems to be unsatisfactory for providing wide band gaps at low and medium frequencies. To improve the isolation performance of wave barriers at low to medium frequencies, a buried PT-WIB consisting of a periodic infilled trench and a wave impedance block barrier has been proposed in this paper. A three-dimensional finite element model has been developed to evaluate the isolation performance of three wave barriers. The influence of the PT-WIB’s parameters on isolation performance has been analyzed. The results indicate that the combined properties of the periodic structure and the wave impedance block barrier can effectively achieve a wide attenuation zone at low and medium frequencies, enhancing the isolation performance for mitigating environmental vibration pollution.

## 1. Introduction

Environmental vibration pollution arising from traffic, machines, and construction blasting has been the subject of increasing concern in recent years, especially train-induced environmental vibration pollution. With the development of rail transportation systems in urban settings, the accompanied vibration from rail systems brings about a negative impact on the surrounding area. The environmental vibrations may become an annoyance issue to surrounding residents in both physiological and psychological aspects [1,2,3,4,5]. Therefore, a lot of attention has been paid to environmental vibration isolation measures. Passive isolation solutions such as open trenches, infilled trenches, and pile barriers [6,7,8,9,10] have been investigated numerically and experimentally. Since these wave barriers may intercept, scatter, or diffract incoming waves, they are generally installed along the propagation paths of the waves in the soil for environmental vibration isolation. However, a broadband attenuation band for environmental vibration has not been achieved through these previous investigations. A relatively broad isolation frequency range is generally required in practical engineering. Thus, it is significant to obtain a specific broadband isolation frequency range.

In recent years, the concept of acoustic metamaterials (AMs) has attracted increasing research attention worldwide due to their peculiar wave dispersion characteristics. The AMs can be regarded as a kind of functional material consisting of identical unit cells with periodic distributions in another medium [11]. The energy of a wave in a particular frequency range can be attenuated through its propagation in the AMs, which is considered to be a band gap or attenuation zone (AZ). The dispersion properties of AMs in physics open up a new horizon for environmental vibration isolation. Subsequently, a large number of investigations, including theoretical analysis, numerical simulations, and experiments, have been conducted to reveal the mechanism of periodic structures in environmental vibration reduction [12,13,14,15,16,17,18,19,20,21]. Huang et al. [15] proposed a layered periodic structure and investigated its frequency zone of vibration reduction using FEM. The simulated AZs were consistent with the results from Bloch theory. Pu and Shi [17] arranged piles in a periodic way and investigated their isolation effects for Rayleigh waves from the perspective of the periodic theory. Huang et al. [19] analyzed the vibration isolation performance of periodic barriers under different excitations through some field experiments. The results indicated that the frequency range of band gaps in the periodic structures can be identified.

Most of the aforementioned studies focused on vibration isolation at medium frequencies. However, the concerning train-induced environmental vibration concentrates on both low and medium frequencies, with a relatively broad range. The dominant frequency of train-induced environmental vibration usually exists in the range of 30–60 Hz. However, the accompanied low-frequency vibration below 10 Hz can travel over a long distance with less attenuation, which may cause more serious impacts on the surrounding area. The attenuation mechanisms of periodic structures are classified as Bragg scattering mechanisms and local resonance mechanisms. For Bragg scattering, the formation of a band gap is based on a complex result of elastic wave reflection and refraction at the interfaces of different materials. It is suitable for medium- and high-frequency vibration reduction, but it has difficulty formulating the low-frequency band gaps [13,14]. The wavelength attenuated by the locally resonant band gap can reach two orders higher than the lattice constant [22], which allows relative material structures to isolate lower-frequency vibration. The local resonance mechanism of periodic structures overcomes the limitations of Bragg scattering theory, and numerous researchers have utilized this mechanism to obtain low-frequency vibration isolation [23,24,25,26]. However, the local resonance mechanism is contradictory to the broadband frequency gaps [26]. 

This paper aims to identify a broadband attenuation zone for environmental vibration at both low and medium frequencies. The wave-impeding block (WIB) is an efficient and cost-effective method for low-frequency vibration reduction. It is usually embedded at a certain depth in the ground under the vibration source and has been widely studied for environmental vibration control [27,28,29,30,31]. The principle of the WIB is to introduce artificially stiffened horizontal layers for the sake of changing the wave propagation mechanism in the ground. Thus, the wave propagation relies on the relationship between the source excitation frequency and the cutoff frequency of the overlaying soil above the WIB. Therefore, a buried PT-WIB (periodic infilled trench–wave impedance block barrier) consisting of a periodic infilled trench and wave impedance block barrier has been proposed in this paper to realize a wide attenuation zone at both low and medium frequencies. A three-dimensional finite element model was developed to analyze the vibration isolation performance of the WIB, periodic infilled trenches, and PT-WIB in both the frequency and time domains. The influence of the different parameters of PT-WIB on the vibration isolation performance is revealed. It is hoped that the present study can be applied to reduce environmental vibration in practical engineering.

## 2. Model and Methods

**Figure 1:**
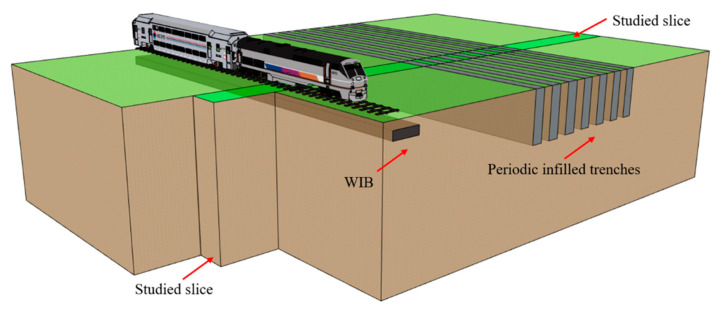
A schematic diagram of the PT-WIB.

The purpose of this paper is to investigate the environmental vibration isolation performance of the proposed PT-WIB. As illustrated in Figure 1, the WIB is installed below the vibration source (under the railway), and the periodic infilled trenches are arranged between the vibration source and the protected objects. A slice of the model includes both the load actions and wave barriers. Thus, the overall effect of the infinite propagation domain can be represented by a slice along the longitudinal direction of the load [16].

**Figure 2:**
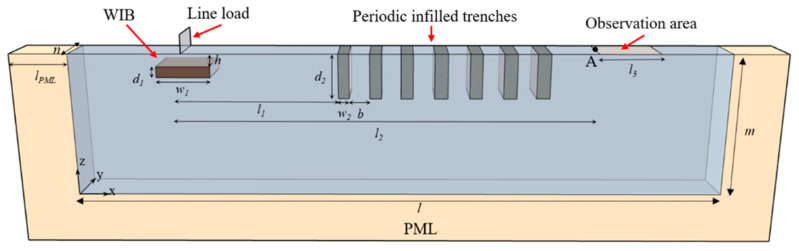
A schematic diagram of the 3D model used for the numerical analysis.

The finite element method using the software COMSOL Multiphysics has been adopted to analyze the isolation performance of wave barriers (WIB, periodic infilled trenches, and PT-WIB) in an elastic half-space. Figure 2 shows the schematic diagram of a 3D model used for the vibration isolation analysis. A pair of periodic boundary conditions (PBCs) were applied to the model in the *y* direction to reduce calculation time. The effectiveness of this simplified model can also be found in related studies [16,17,32,33]. The dimensions of the considered model, *l* × *m* × *n*, are 40 m, 20 m, and 1 m, respectively. The widths of the WIB and infilled trench are *w*_1_ and *w*_2_; the depths of the WIB and infilled trench are *d*_1_ and *d*_2_, respectively; *l*_1_ is the distance from the vertical harmonic line source to the first row of infilled trenches; *l*_2_ is the distance from the vertical harmonic line source to the observation area of the vibration response; *l*_3_ is the length of the observation area for the vibration response; and *h* is the embedded depth of the WIB. The spacing of the periodic infilled trenches is *b*. The materials used for the periodic infilled trenches and WIB are geofoam and concrete, respectively. Low-density geofoam exhibits significant energy dissipation capacity and excels as a vibration isolation infill material, offering numerous advantages over alternative infill materials. This superiority stems from its lightweight nature, economic viability, and exceptional isolation efficiency. Concrete, renowned for its high-strength properties, is frequently employed as a widely used material for WIB. The properties of the soil and wave barrier materials were considered based on some previous studies [18,34,35], as shown in Table 1. It was assumed that the materials of the soil and wave barriers are elastic, isotropic, and homogeneous. The damping factor of the soil was 0.05. The perfectly matched layer (PML), as an effective absorption boundary condition, was added on the left, right, and bottom of the considered model to simulate semi-infinite media in the frequency domain analysis [36]. The velocity of the Rayleigh waves *V*_R_ was calculated in view of the propagation of surface waves in the soil, as follows:

(1) $$V_{R}=\frac{\sqrt{E/p}}{\sqrt{2(1+v)}}\cdot \frac{0.87+1.12v}{1+v},$$

where *E*, *p*, and *v* represent the Young modulus, density, and Poisson ratio of the soil, respectively. The Rayleigh wavelength *λ_R_* was calculated to be 2 m at a frequency of 45 Hz.

The amplitude reduction ratio *A_R_*, which is represented as the ratio between surface displacement amplitude with and without a wave barrier, was used to assess the vibration isolation performance of wave barriers, and it can be expressed as [6]:

(2) $$A_{R\;}=\frac{u_{y1}}{u_{y0}},$$

where *u_y_*_1_ and *u_y_*_0_ contribute the surface displacement amplitude with and without a wave barrier, respectively. The area-averaged frequency response function can be described as:

(3) $$\bar{A_{R}}=\frac{1}{nl_{3}}\int_{0}^{n}\int_{0}^{l_{3}}A_{R}\mathrm{dxdy}.$$

The wave attenuation through the trenches would appear when $\bar{A_{R}}$ is less than 1. Thus, $\bar{A_{R}}$ was adopted to assess the vibration isolation performance in relation to the barriers.

**Figure 3:**
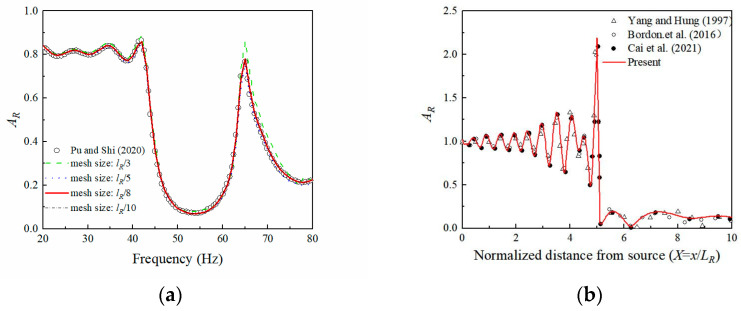
Comparative study for vibration isolation: (**a**) periodic geofoam-filled trenches; (**b**) open trench. Retrieved from refs. [20,34,37].

A mesh size convergence study was carried out to verify the accuracy of the numerical model. The present numerical simulation results in respect to four different mesh sizes are compared with the solutions of [18], as shown in Figure 3a. It is indicated that the simulation results of vibration isolation by periodic geofoam-filled trenches converged to the exact solution as the mesh size decreased. A tetrahedral mesh size up to *λ_R_*/8 was sufficient to satisfy the calculation requirements, and further reduction of the mesh size would not affect the accuracy of the results [36]. Hence, the model can be discretized into tetrahedral elements with a size of *λ_R_*/8 for subsequent studies. For the sake of substantiating the numerical model further, the vibration isolation performance of an open trench with 1 *λ_R_* depth and 0.1 *λ_R_* width was utilized as a reference, which was located 5 *λ_R_* away from the excitation source. It can be seen from Figure 3b that the present results are identical to the previous research results of Yang and Huang [34] and Bordon et al. [37]. As a consequence of the comparison research, the present grid precision is appropriate for numerical simulations.

## 3. Results and Discussions

In order to validate the isolation effectiveness of the PT-WIB, extensive analyses were conducted to investigate the vibration isolation performance of three kinds of wave barriers (WIB, periodic infilled trenches, and PT-WIB) under different excitations in both the frequency domain and time domain. Whereafter, a parametric analysis is proposed to investigate the behavior of the PT-WIB.

### 3.1. Isolation Characteristics of Three Kinds of Wave Barriers in the Frequency Domain

For the sake of illustrating the vibration isolation performance of these wave barriers in a more direct way, the parameters of periodic infilled trenches are referred to in previous studies [18] as: *d*_2_ = 2 m, *w*_2_ = 0.3 m, *b* = 0.7 m. The number of rows for infilled trenches is seven. The parameters of the WIB for the analysis are *d*_1_ = 0.3 m, *w*_1_ = 3 m, *h* = 0.45 m. The PT-WIB is a combination of the WIB and periodic in-filled trenches; thus, the parameters of the PT-WIB are the same as those of the above wave barriers. In addition, *l*_1_ = 10 m, *l*_2_ = 20 m, and *l*_3_ = 6 m are chosen.

**Figure 4:**
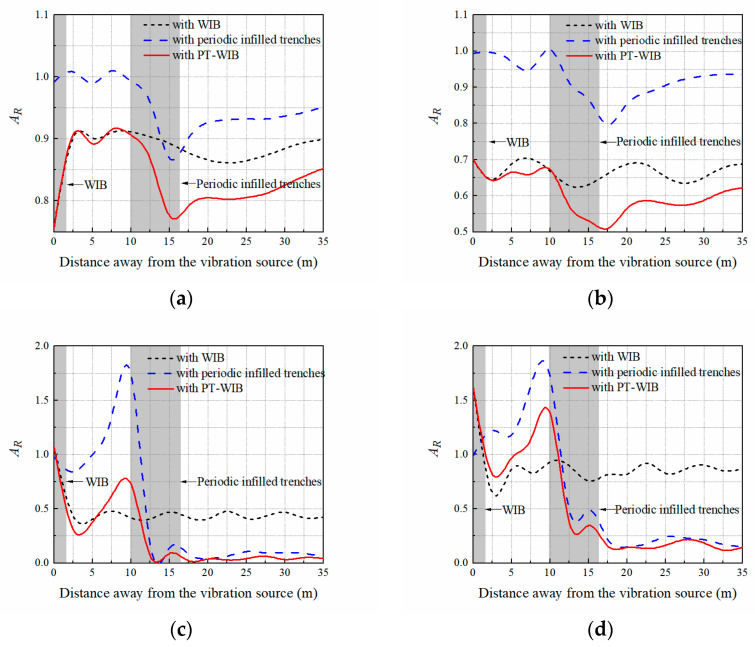
Vibration isolation performances with respect to three kinds of wave barriers at different fixed frequency excitation: (**a**) 8 Hz; (**b**) 15 Hz; (**c**) 55 Hz; (**d**) 80 Hz.

**Figure 5:**
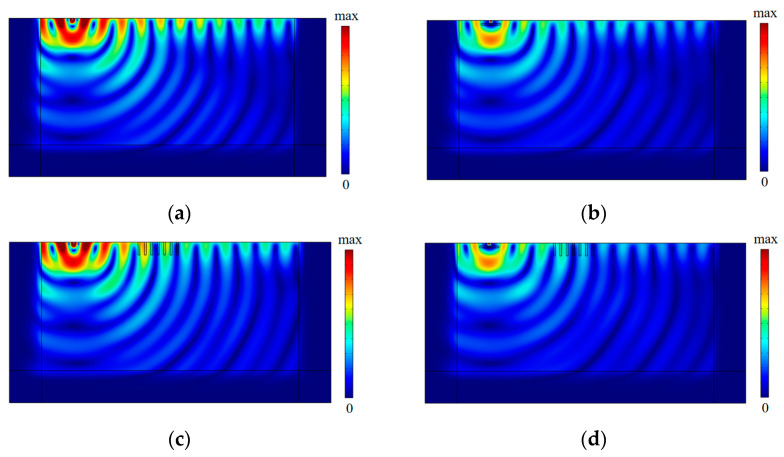
The nephogram of vertical displacement amplitude field at *f* = 15 Hz: (**a**) without wave barriers; (**b**) with WIB; (**c**) with periodic infilled trenches; (**d**) with PT-WIB.

Figure 4a,b show the vibration isolation performance with respect to three kinds of wave barriers at low excitation frequencies (8 Hz and 15 Hz). The isolation effectiveness of the PT-WIB for low-frequency vibrations was better than that of the WIB and periodic infilled trenches, especially in the region behind the trenches (*x* > 10 m). The vibration isolation effectiveness of periodic infilled trenches is dissatisfactory at low excitation frequencies. This is because wavelength is longer at a lower frequency, and shallow trenches have difficulty achieving acceptable isolation performance. The results also show that the WIB had the advantage of reducing the low-frequency vibration, which is consistent with other investigation results [29,30,31]. The nephogram of the vertical displacement amplitude field with three different kinds of wave barriers at the excitation frequency of 15 Hz is shown in Figure 5.

**Figure 6:**
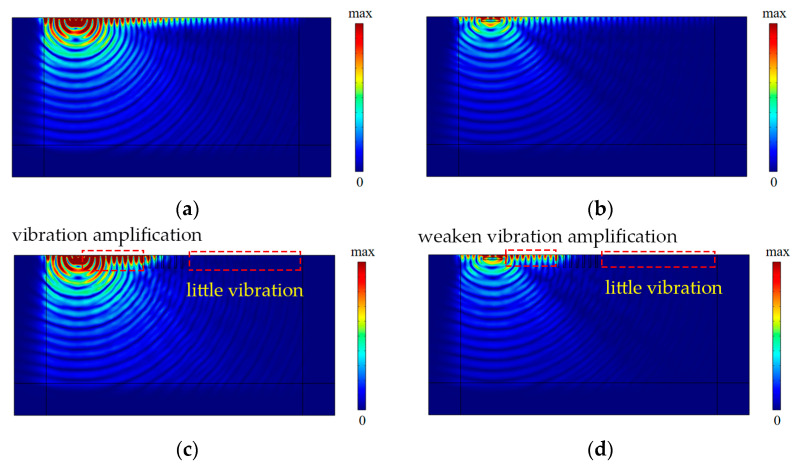
The nephogram of vertical displacement amplitude field at *f* = 55 Hz: (**a**) without wave barriers; (**b**) with WIB; (**c**) with periodic infilled trenches; (**d**) with PT-WIB.

Figure 4c,d compare the vibration isolation performances with respect to three kinds of wave barriers at medium- and high-excitation frequencies (55 Hz and 80 Hz). It can be found that the vibration isolation effectiveness of the periodic infilled trenches was rather high in the region behind the periodic infilled trenches at medium- and high-excitation frequencies. This is because the designed periodic infilled trenches could isolate the surface waves of the attenuation zone (45–62 Hz) effectively, and there was a region of evanescent surface waves above the attenuation zone, namely, leaky surface modes [18]. Thus, the surface waves of 55 Hz and 80 Hz could also be isolated effectively, whereas some vibration amplification was observed in front of the periodic infilled trenches on account of the vibration reflection at the interface of the geofoam and the ground. Whereas WIB performed better in the area in front of the trenches (*x* < 10 m), its isolation effectiveness was inferior to that of the periodic infilled trenches in the region behind the trenches (*x* > 10 m). It should be noted that the PT-WIB combined the vibration isolation advantages of both the WIB and the periodic infilled trenches. It could isolate specific surface waves of the attenuation zone due to the presence of the periodic infilled trenches and take advantage of the WIB to weaken the vibration amplification in the front region of the periodic infilled trenches. The difference in vibration isolation for these kinds of wave barriers at an excitation frequency of 55 Hz can also be observed in Figure 6.

**Figure 7:**
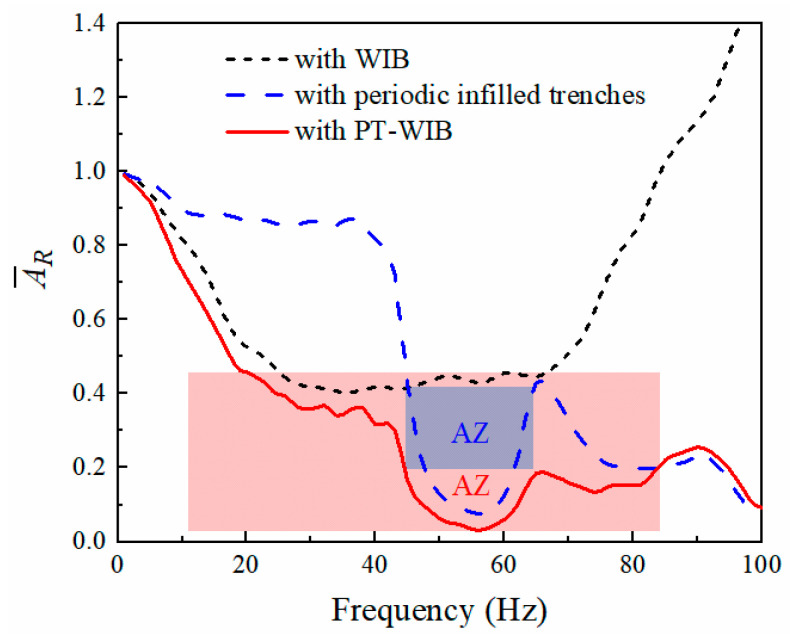
Vibration isolation performance with frequency with respect to three kinds of wave barriers.

Figure 7 illustrates vibration isolation performance with respect to three kinds of wave barriers in the frequency domain. The observation area for vibration response is shown in Figure 2. It can be noticed from Figure 7 that the vibration isolation performance of the periodic infilled trenches was effective at medium- and high-excitation frequencies, especially in the attenuation zone (45–62 Hz). The WIB was more efficient in reducing low- and medium-frequency vibration, but it was limited in isolating high-frequency vibration. The PT-WIB can reduce low-frequency vibration as well as the isolation of surface waves in some specific ranges, which are expected to be the frequency band gaps for the periodic structure. Thus, a broadband attenuation zone for the vibration could be achieved, which is difficult to achieve by other single measures. Moreover, the countermeasure was easy to apply in practical engineering.

### 3.2. Isolation Characteristics of Three Kinds of Wave Barriers in the Time Domain

**Figure 8:**
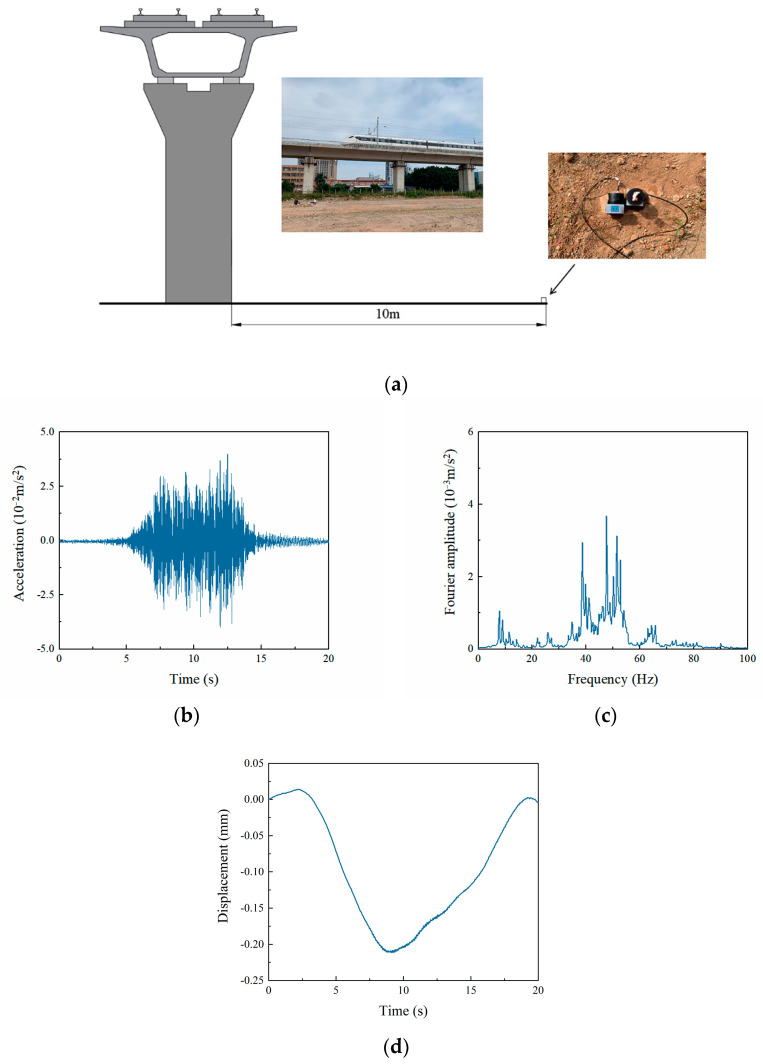
Vibration response in the field caused by the intercity railway: (**a**) field measure; (**b**) vertical acceleration record; (**c**) Fourier spectrum; (**d**) the corresponding displacement excitation.

In this section, the isolation effectiveness with respect to these three kinds of wave barriers is investigated in the time domain. A field test to measure traffic-induced environmental vibrations was conducted in Dongguan (Guangdong Province, China), as shown in Figure 8a. The type of daily operating elevated intercity train was CRH6A. The 941B-type ultra-low accelerometers were used to collect the accelerometer signal, and the INV3062-type vibration signal acquisition instrument was used to process and analyze the signals. The location of the test was 10 m away from the pier in a field. Figure 8b,c shows the measured acceleration record and the corresponding Fourier spectrum when a train ran by at approximately 100 km/h, respectively. The measured acceleration data were integrated twice to acquire the displacement excitation, as described in Figure 8d, which was applied at the load location shown in Figure 2. The duration of the railway excitation was 20 s; the dynamic sub-step was 20,000; and the time step of integration was approximately 0.001 s.

**Figure 9:**
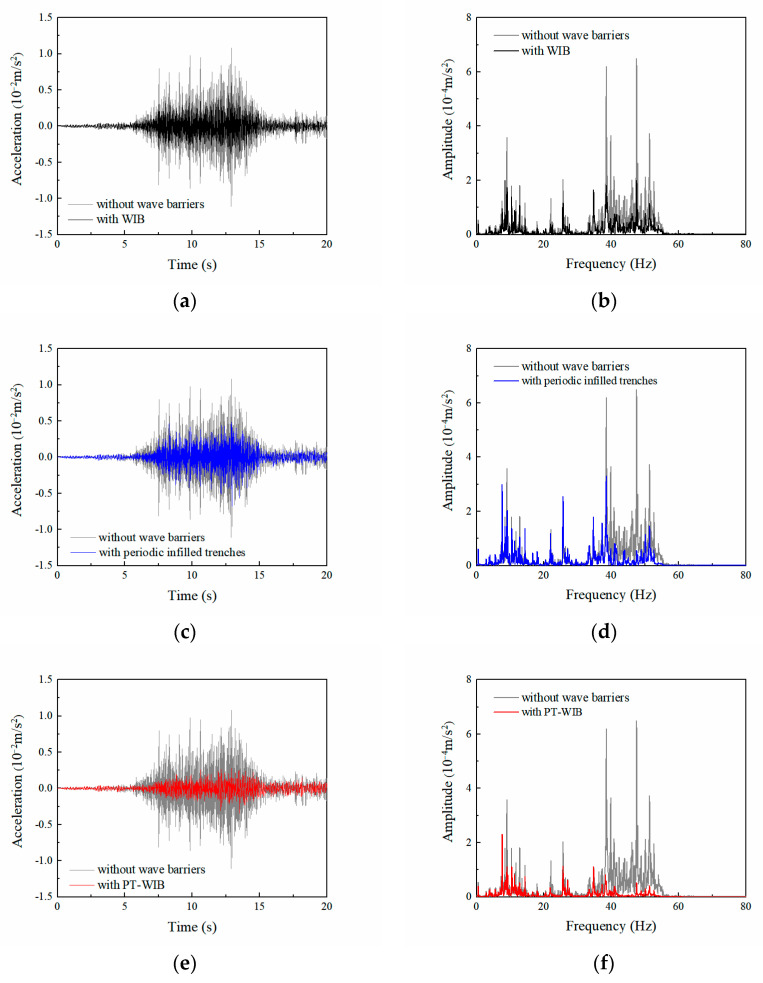
Vertical acceleration responses at the detection point and corresponding Fourier spectrum with and without barriers: (**a**,**b**) with and without WIB; (**c**,**d**) with and without periodic infilled trenches; (**e**,**f**) with and without PT-WIB.

The geometries and arrangements of three types of wave barriers were the same as described in Section 3.1. PMLs are typically not employed in time-domain analysis. Instead, in the model, a low-reflective boundary condition was implemented to effectively mitigate the reflection of vibration waves during time-domain analysis. Figure 9 shows vertical acceleration responses and the corresponding Fourier spectrum at the detection point A (*x* = 20 m) with three kinds of wave barriers and without barriers subjected to railway excitation. It can be seen that the PT-WIB provided better isolation performance than other wave barriers; the acceleration amplitude with the PT-WIB was reduced by 74.8% when compared to that without a wave barrier. And the acceleration amplitudes with the WIB and periodic infilled trenches were reduced by 54.6% and 52.4%, respectively. Meanwhile, it can be observed from the Fourier spectra of the PT-WIB that vibrations were attenuated over the whole observed frequency range. The analysis results in the time domain reveal the feasibility of isolating environmental vibration with PT-WIB in practice.

### 3.3. Parametric Study of the PT-WIB

The vibration isolation performances of three different wave barriers have been analyzed in both the frequency domain and the time domain. The proposed PT-WIB shows the advantage of achieving a wide attenuation zone in environmental vibration isolation. A parametric study was carried out to analyze the influence of these effects on the vibration isolation performance of the PT-WIB. A large number of investigations, including theoretical analysis, numerical simulations, and experiments, have been conducted to reveal the mechanism of periodic structures in environmental vibration reduction, and a reasonable design process for isolating vibration in a specific frequency range has been proposed. The purpose of this study was to extend the attenuation zone and improve the vibration isolation performance by introducing the WIB into the periodic infilled trenches. Therefore, only the influences of parameters associated with the WIB (width, thickness, embedded depth, and distance from the source) on the isolation performance were analyzed. The parameters of the periodic infilled trenches remain unchanged, and the parameters (*d*_2_ = 2 m, *w*_2_ = 0.3 m, *b* = 0.7 m) are referring to [18]. The observation area for the vibration response is shown in Figure 2.

**Figure 10:**
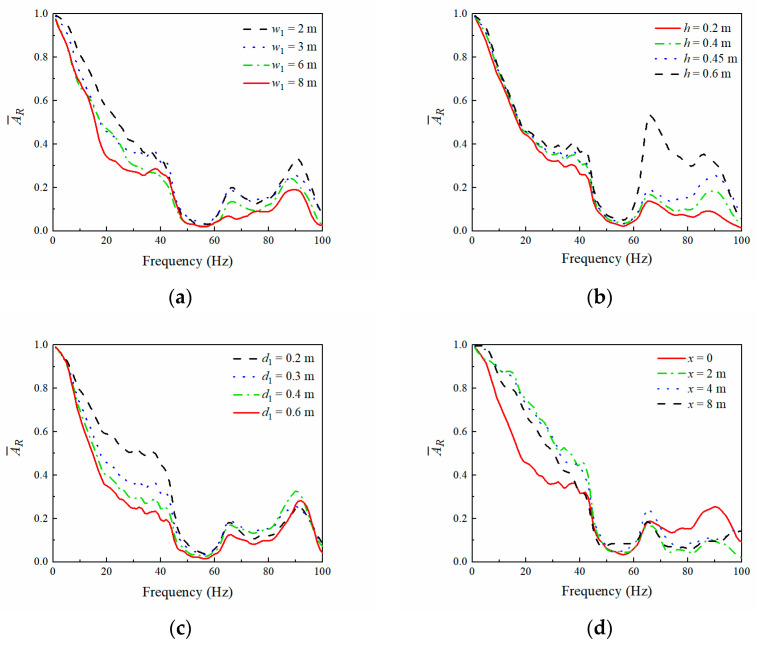
Influence of WIB’s parameters on ($\bar{A_{R}}$): (**a**) width (*d*_1_ = 0.3 m, *h* = 0.45 m, *x* = 0); (**b**) thickness (*w*_1_ = 3 m, *h* = 0.45 m, *x* = 0); (**c**) embedded depth (*w*_1_ = 3 m, *d*_1_ = 0.3 m, *x* = 0); (**d**) distance from excitation source of WIB (*d*_1_ = 0.3 m, *w*_1_ = 3 m, *h* = 0.45 m).

Figure 10a,b show that the average amplitude reduction ratio $\bar{A_{R}}$ of the observation area varied with the WIB’s width and thickness, respectively. It can be observed that the vibration isolation effectiveness of the PT-WIB increased with the width and thickness of the WIB, especially in the frequency region outside the attenuation zone (45–62 Hz) of the periodic infilled trenches. The vibration isolation effectiveness increased with a decrease in the embedded depth of the WIB, as shown in Figure 10c. The embedded depth of the WIB varied from 0.2 m to 0.6 m, and there was an obvious increase in vibration isolation effectiveness, particularly in the high-frequency region. The effect of the distance from the excitation source of the WIB on $\bar{A_{R}}$ is shown in Figure 10d. It is evident that the WIB installed below the vibration source (*x* = 0) was more efficient for isolating low- and medium-frequency waves. A better performance for high-frequency vibration isolation can be realized by placing the WIB in the appropriate position between the vibration source and periodic infilled trenches.

**Figure 11:**
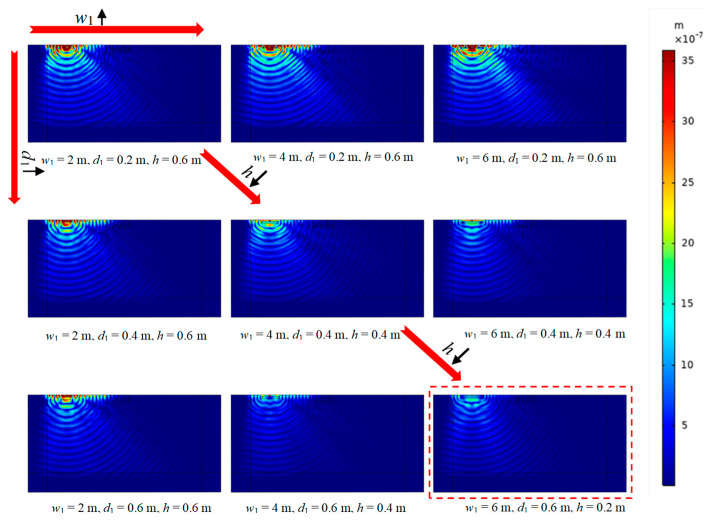
The nephogram of vertical displacement amplitude field for the PT-WIB with different parameters (width, thickness, and embedded depth of WIB) at *f* = 55 Hz. (The direction indicated by the red arrow represents the direction of parameter change. The nephogram within the red dashed line box visually demonstrates that the most significant vibration reduction effect is achieved when the parameters are combined in a specific manner.)

Furthermore, the nephogram of the vertical displacement amplitude field for the PT-WIB with respect to different parameters (WIB’s width, thickness, and embedded depth) is illustrated in Figure 11 in order to demonstrate these parameters’ effects on the vibration isolation throughout the region in a more direct way. Since the effects of different parameters on the observation area after the periodic infilled trenches have been discussed above, attention has been paid to the region before the periodic infilled trenches (0 < *x* < 10) here. The *w*_1_ varied from 2 m to 6 m, the *d*_1_ varied from 0.2 m to 0.6 m, and the *h* varied from 0.2 m to 0.6 m. It can be seen from Figure 11 that the embedded depth of the WIB played a significant part in the vibration isolation of the region before the periodic infilled trenches. The vibration isolation performance could be distinctly improved with a decrease in the embedded depth of the WIB. Furthermore, the first row and column of the nephogram in Figure 11 reveal that the isolation performance improvement of the PT-WIB was not so obvious when increasing the width and thickness of the WIB. This indicates that decreasing the embedded depth of WIB is the most effective way to improve the vibration isolation performance of the PT-WIB.

### 3.4. The Application of PT-WIB in Layered Ground

**Figure 12:**
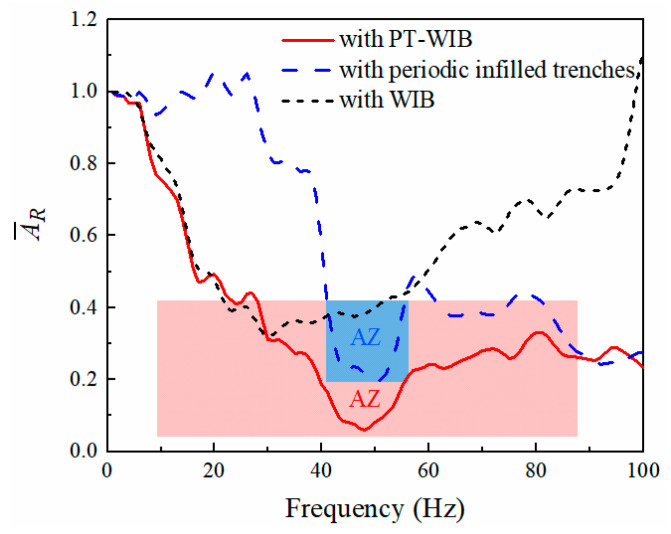
Vibration isolation performance with respect to three kinds of wave barriers in layered-soil medium.

**Figure 13:**
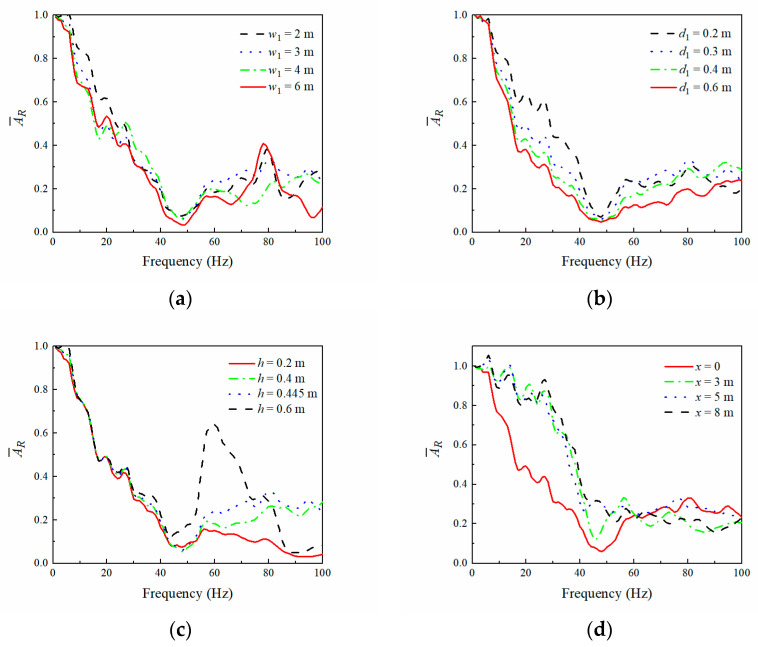
Influence of WIB’s parameters $\bar{A_{R}}$ in layered-soil medium: (**a**) width (*d*_1_ = 0.3 m, *h* = 0.45 m, *x* = 0); (**b**) thickness (*w*_1_ = 3 m, *h* = 0.45 m, *x* = 0); (**c**) embedded depth (*w*_1_ = 3 m, *d*_1_ = 0.3 m, *x* = 0); (**d**) distance from excitation source of WIB (*d*_1_ = 0.3 m, *w*_1_ = 3 m, *h* = 0.45 m).

In practice, the soil is generally layered rather than single-layered. The soil medium with layered soil [27] was adopted to replace the single-layer soil. The properties of the layered soil are referred to in [27]. The vibration isolation performances with respect to these three wave barriers in the layered soil were compared. The geometric dimensions, number of rows, and material properties of the periodic infilled trenches and WIB are the same as those described in Section 3.1. It can be observed from Figure 12 that the vibration isolation performance of PT-WIB is excellent in the layered-soil medium, which is consistent with the results in Section 3.1. Simultaneously, a parametric study, which is similar to Section 3.3, was carried out, as shown in Figure 13. It indicates that the influence of different WIB parameters on vibration isolation is similar to that of Section 3.3.

## 4. Conclusions

In rail transit, vibration is the main source of environmental pollution. This paper proposes a buried PT-WIB consisting of periodic infilled trenches and a wave-impedance block barrier to achieve a broadband attenuation zone for rail transit vibration isolation. The main conclusions are summarized as follows:

- The WIB is positioned beneath the railway tracks, while periodic infilled trenches are strategically placed between the railway and the protected buildings. In situations where the vibration isolation requirements cannot be met solely by the periodic trenches, typically due to the limited effectiveness of a narrow band gap at low and medium frequencies, the newly proposed PT-WIB offers a practical and viable solution. This innovative approach demonstrates the convenience and feasibility of creating a broadband attenuation zone, effectively addressing the limitations encountered in traditional setups.
- The occurrence of vibration amplification phenomena is observed in the vicinity of periodic infilled trenches and is primarily attributed to wave reflections at the interface between the geofoam and the ground. However, the implementation of the WIB effectively mitigates these vibration amplifications. Consequently, the newly proposed PT-WIB offers a notable advantage by providing a relatively consistent and stable environmental vibration isolation performance throughout varying distances from the vibration source.
- Although an increase in the width and thickness of the WIB can improve the vibration isolation performance of the PT-WIB, a decrease in the embedded depth of the WIB is a more effective way to improve the vibration isolation performance of the PT-WIB. Moreover, the PT-WIB can also be applied to a layered ground for the improvement of vibration isolation performance.

## Figures and Tables

**Table 1:** The material parameters of the soil and the barriers.

Material	Young Modulus *E* (MPa)	Poisson Ration *v*	Density *p* (kg/m^3^)
Soil	46	0.25	1800
Geofoam	37	0.32	60
Concrete	25,500	0.20	2500

## Data Availability

Data are available upon request.
